# Total lipid prediction in single intact cocoa beans by hyperspectral chemical imaging

**DOI:** 10.1016/j.foodchem.2020.128663

**Published:** 2021-05-15

**Authors:** Nicola Caporaso, Martin B. Whitworth, Ian D. Fisk

**Affiliations:** a Division of Food Sciences, University of Nottingham, Sutton Bonington Campus, LE12 5RD, UK; b Campden BRI, Chipping Campden, Gloucestershire GL55 6LD, UK

**Keywords:** *Theobroma cacao*, Hyperspectral imaging, Near-infrared spectroscopy, Chemical imaging, Total lipid quantification, Cocoa quality assessment, Cocoa nibs, Cocoa butter

## Abstract

This work aimed to explore the possibility of predicting total fat content in whole dried cocoa beans at a single bean level using hyperspectral imaging (HSI).

170 beans randomly selected from 17 batches were individually analysed by HSI and by reference methodology for fat quantification. Both whole (i.e. in-shell) beans and shelled seeds (cotyledons) were analysed. Partial Least Square (PLS) regression models showed good performance for single shelled beans (R^2^ = 0.84, external prediction error of 2.4%). For both in-shell beans a slightly lower prediction error of 4.0% and R^2^ = 0.52 was achieved, but fat content estimation is still of interest given its wide range. Beans were manually segregated, demonstrating an increase by up to 6% in the fat content of sub-fractions.

HSI was shown to be a valuable technique for rapid, non-contact prediction of fat content in cocoa beans even from scans of unshelled beans, enabling significant practical benefits to the food industry for quality control purposes and for obtaining a more consistent raw material.

## Highlights

• Quantitative calibrations were built from shelled and in-shell single cocoa beans by HSI.
• The fat content of commercial batches of cocoa beans varies by up to 15% within batches.
• HSI prediction of the total lipid content was successful for shelled and unshelled beans.
• Segregation using HSI fat calibration enhanced cocoa bean fat content by 6%.

## 1 Introduction

Cocoa beans have high commercial importance worldwide due to their use as the primary ingredient in chocolate. One of the prominent quality factors for cocoa is its lipid content (Fowler, 2008). Fat represents approximately half of the cocoa bean’s weight and is used to produce cocoa butter, which is one of the most valuable products of the bean and a strong determinant of its market price (Afoakwa, 2017).

Several methods are available for the analysis of fat content in food products (AOAC, 2006, ElKhori et al., 2007, Möller, 2010, ISO, 2014, ISO, 2019). The Soxhlet extraction method is one of the most common analytical approaches, and is based on the gravimetric determination of crude fat, followed by solvent extraction. This method has often been reported to give lower results than other methods such as those where digestion or hydrolysis is used. Indeed, bound fats or those naturally emulsified are not measured by Soxhlet. Conversely, methods based on ether extraction tend to overestimate fat content. Other methods for fat analysis are based on acid hydrolysis, followed by saponification of the fats and esterification to give methyl esters of the fatty acids, which can be analysed by gas-chromatography. Despite the accuracy of these methods, they are very time consuming, only are effective on relatively large batches of samples and involve the use of hazardous chemicals. Additionally, they are destructive methods, thus not allowing using the samples for further analyses or for their use in processing or assessing the variability of biochemical composition across within the batch (ISO, 2019).

Rapid non-destructive techniques for the analysis of major food constituents include Near-Infrared (NIR) spectroscopy, mid-infrared (MID) spectroscopy and Raman spectroscopy (Osborne et al., 1993, Caporaso et al., 2018, Türker-Kaya and Huck, 2017, Xu et al., 2020). Although typically used to measure the average composition of bulk samples, hyperspectral imaging (HSI) enables spectra to be obtained for each pixel in an image, enabling spatial variations in composition to be measured (Caporaso, Whitworth and Fisk, 2018). When applied to food product characterisation, HSI can provide information on chemical composition or other properties, as well as spatial information, such as distribution across a sample (Gowen et al., 2007, Elmasry et al., 2012).

Recent works reported on the successful prediction of bioactive compounds in cocoa bean husks using conventional NIR spectroscopy (Hernández-Hernández et al., 2020), and using hyperspectral imaging (HSI) to predict the antioxidant activity, total phenolic content and the fermentation index of whole cocoa beans (Caporaso, Whitworth, Fowler and Fisk, 2018). NIR spectroscopy has also been applied to quantify the amount of cocoa shell in cocoa powder due to contamination from processing, and showed good results (Burns and Ciurczak, 2007, Workman and Weyer, 2012), e.g. Quelal-Vasconez et al. (2019) reported that NIR successfully distinguished contamination above 5%, with a root mean square error of prediction (RMSEP) of 2.43%. Hyperspectral imaging has also recently been applied for fast authentication of two cocoa hybrids, e.g. Cruz-Tirado et al. (2020) reported that the classification models based on SVN and PLS-DA had promising results, with classification errors ranging from 4 to 34%.

FT-NIR spectroscopy has been applied for fat quantification in cocoa beans, by scanning the samples as ground, showing excellent prediction performance (Teye and Huang, 2015). A similar technique was applied by Sunoj et al. (2016), and Teye et al. (2015) for the prediction of fermentation index, pH and polyphenols in cocoa beans, scanned as ground material. However, since these were bulk methods, they cannot detect the distribution within batches as they can only measure the average content. There is a lack of studies on the prediction of lipids, which have the largest economic implication for this commodity.

NIR spectroscopy has been proven to be effective for fat quantification in several food products including cocoa (Veselá et al., 2007, Vines et al., 2005). Kays, Archibald and Sohn (2005) analysed a diverse set of intact cereal products by NIR spectroscopy, reporting an average SECV of 1.18% and R^2^ = 0.98, using gravimetric determination by extracting the fat with petroleum ether as the reference method. Wang et al. (2006) built calibrations for rice grains and flour, as batch, by NIR spectroscopy and reported good results, with R2 values ranging from 0.79 to 0.91, and RMSE from 0.08 to 0.16%.

Previous literature using NIRS for the analysis of total fat in cocoa beans used ground material, thus only the average fat content was predicted, not allowing any investigation on the single bean variability. For example, Fourier-Transform NIR (FT-NIR) has been successfully applied in the spectral region 10,000–4,000 cm^−1^ to investigate the total fat content in ground shelled cocoa beans (i.e. ground cocoa nibs) (Teye & Huang, 2015). The fat content ranged from 51.3 to 68.0% and the calibration and prediction R^2^ values were 0.93–0.98 and 0.92–0.97 respectively, depending on the different PLS regression models used. The prediction error was RMSECV = 0.01–0.02% and RMSEP ~ 0.02%. Fifty samples were used for the calibration dataset and 30 for prediction. Despite the good calibration performance, no information on the bean-to-bean variability was reported, and more importantly, it should be noted that this method still requires the removal of the cocoa bean shell and grinding of the resulting nibs to the required dimensions.

Veselá et al. (2007) compared NIR (1100–2500 nm) and FT-IR (2500–25,000 nm) for the prediction of fat, nitrogen and moisture content in cocoa powder. Fat content exhibits a wide range, i.e. 2.4–22.0% as expected, as cocoa powder is made by grinding cocoa nibs and removing some of the fat. The NIR prediction model for this constituent achieved R^2^ = 0.96 and RMSECV = 7.0%. By FT-IR, the prediction model had R^2^ = 0.94 and RMSECV = 10.4%.

NIRS has also been reported to predict total fat content in shelled cocoa beans by Álvarez et al. (2012). The authors applied reflectance spectroscopy in the region 780–2500 nm to evaluate fat, caffeine, theobromine and epicatechin content. On a fat content ranging from 46 to 64%, the R^2^ value was 0.94, SECV = 0.89%, and RPD = 3.4. Additionally, the fat content of Criollo types was generally reported to be lower than other cocoa types like Forastero and Trinitario.

The only paper found in the literature on whole cocoa bean analysis using FT-NIR was by Sunoj et al. (2016). FT-NIR (800–2778 nm) was used to scan whole cocoa beans obtained from one batch fermented at different fermentation times, from 1 to 6 days. Prediction models were built for predicting polyphenol content, pH and fermentation index. However, no indication about fat content prediction was reported.

Despite the efforts to build prediction models for important quality attributes of cocoa, traditional NIR instruments and the approaches used so far are not capable of investigating single cocoa bean variability while rapidly predicting fat content in a non-destructive manner. Moreover, and more importantly, existing NIR approaches require cocoa beans to be unshelled and ground, which is a time consuming manual process.

Several studies have shown the potential of HSI for fat prediction in grains, nuts or seeds, especially for single objects. A recent publication demonstrated its application for green coffee beans (Caporaso et al., 2018). Jin et al. (2016) applied HSI using two detectors, working at 400–1000 and 1000–2500 nm, to measure oil content in single peanuts. The authors used five varieties, sampling 30 nuts per batch. The performance of the PLS regression models had prediction R^2^ values of 0.67–0.92, and error (RMSEP) of 0.21–0.42%.

The literature is lacking in relation to the use of HSI for non-destructive prediction of lipid content of whole cocoa beans, or to investigate the distribution of fat content within the beans. A recent paper investigated the feasibility of HSI to predict fermentation index, antioxidant activity and phenolic content in cocoa beans (Caporaso, Whitworth, Fowler and Fisk, 2018), but lipid content was not assessed and all beans were shelled. Lipid content is the most critical factor for cocoa bean quality assessment and in defining its commercial price. Therefore, the aim of the present work was to investigate the feasibility of HSI to non-destructively analyse unshelled and shelled cocoa beans on a single bean basis in order to predict total fat content and its intra-bean distribution.

## 2 Materials and methods

### 2.1 Samples and reagents

Seventeen samples of commercial cocoa beans were obtained from Ghana, Indonesia, Ivory Coast, Nigeria, Ecuador, Cameroon, Brazil, Venezuela, and Mexico, including all the major cocoa producing countries. Ten batches were of Forastero type, six were of Trinitario type and one was of unknown type. Ten beans were randomly selected from each of the 17 batches and analysed by HSI without any further treatment. The samples were scanned by HSI, first as un-shelled, then after they were manually shelled. Once the HSI acquisition was performed, the shelled cocoa beans were manually ground using a mortar and pestle. The ground samples were then stored in closed Eppendorf tubes at −20 °C prior to chemical analysis.

As a verification of the performance of the calibration, an additional small batch was scanned by HSI, the model was applied on this data and single seeds were manually selected based on the predicted total fat content, categorised as low-fat content, high-fat content, remaining seeds. These fractions were analysed by the reference method to measure the average lipid content of each fraction.

### 2.2 Hyperspectral imaging analysis

A SWIR HSI system described in Caporaso, Whitworth, Fowler and Fisk (2018) was used. The system consisted of an instrument provided by Gilden Photonics Ltd. (Glasgow, U.K.) and includes a SWIR spectral camera (Specim Ltd., Oulu, Finland) containing a cooled 14 bit 320 × 256 pixel HgCdTe detector and N25E spectrograph providing 256 spectral bands over a wavelength range of ~ 980–2500 nm with a spectral resolution of about 6 nm. Only the final 240 spectral bands contained useful data, and the first 16 bands were excluded. The acquisition was based on a push-broom approach, with the sample placed at a distance of 220 mm and using 31 mm focal length lens. The samples were scanned while moving at a speed of 10.9 mm s^−1^ to provide square pixels. The illumination was based on two 500 W incandescent lamps.

SpectralCube 3.0041 software (Specim) was used to control the moving stage on which the samples were placed, and the camera acquisition parameters. The dark reference was acquired by recording ~ 100 frames after closing the camera shutter after each data acquisition, while the white reference was acquired by scanning a white PTFE reference material with ~ 100% reflectance.

Samples of single cocoa beans were analysed by HSI as unshelled (i.e. whole, as received) or shelled unground beans (i.e. cotyledons or nibs). Ten cocoa beans at a time were placed on a moveable plastic stage and scanned using the push-broom approach. Details on the instrument, image acquisition, processing and hypercube data management have been previously described in Caporaso, Whitworth, Fowler and Fisk (2018). Each cocoa bean was scanned on both sides, so that the final number of average spectra for the prediction models was 340. Cocoa beans were manually de-shelled and scanned again by HSI. They were then individually ground using a manual mortal, yielding approximately 1 g material for each bean. The samples were then stored at −20 °C, in readiness for reference analyses. The average spectra for each cocoa bean were exported for statistical analysis.

### 2.3 Total fat analysis

Fat content reference determination was carried out using Nuclear Magnetic Resonance (NMR), which is known to have very good precision for total lipid assessment (McManus and Horn, 2004), and has been recently applied for similar experiments on other granular food commodities (Caporaso, Whitworth, Grebby and Fisk, 2018). Single cocoa beans were manually shelled and stored at −20 °C for at least one hour to obtain fat crystallisation. The ground material was analysed by a CEM Smart Trac II Moisture and Fat analyser (CEM Microwave Technology Ltd., Buckingham, UK), which has a resolution of 0.01%. Fat content was either expressed on “as is” basis, or on a dry matter basis (dmb), based on bean moisture measurements made with a CEM microwave moisture analyser. The same reference method was successfully applied in our previous work on single green coffee bean to analyse total lipid content (Caporaso, Whitworth, Grebby and Fisk, 2018).

To verify the accuracy and repeatability of the method, one batch of ground cocoa beans was analysed in 10 replicates. The analytical error, expressed as standard deviation for 10 replicate measurements (SD) was 0.81% (“as is”), with a coefficient of variation (CV) of 1.19%.

### 2.4 Colour measurement

The colour of individual unshelled cocoa beans and of ground cocoa nibs was determined using a DigiEye imaging system (VeriVide, Leicester, UK). Colour of in-shell cocoa beans and cocoa nibs (as ground) was assessed for each seed within the CIE L* a* b* colour space. Samples were placed in the DigiEye chamber under standard light conditions and colour measurements were analysed using the provided software. The instrument was standardised for white balance and uniformity, and colour was calibrated using a reference colour chart. Images were acquired on both sides of the in-shell beans and the average colour was calculated, while one picture of the ground material was taken for sample (individual cocoa bean).

### 2.5 Data treatment and statistical analysis

The spectral data exported for single cocoa beans (whole, i.e. ‘in-shell’, and shelled) were analysed using the Unscrambler 10.3 software (CAMO, Norway). Spectra acquired from two sides of each cocoa bean (whole and shelled) were randomly split into calibration and validation datasets, using an holdout approach (70:30 ratio), and making sure that spectra from the same bean were all included in either the calibration or validation set. The splitting into calibration and validation datasets was performed by randomised sampling from the total cocoa beans of 170 samples. PLS regression calibrations were evaluated based on the coefficient of regression (R^2^) and the root mean square error of calibration (RMSEC), cross validation (RMSECV) and prediction (RMSEP), as well as using the Ratio to Performance Deviation (RPD), which is defined as the ratio between the measured standard deviation and the prediction error.

### 2.6 Visualisation of chemical images

Once established, the best PLS regression prediction model was applied to the hypercubes, by exporting and using the weighted beta-coefficients. The calibration was applied in two ways; at single pixel level, by multiplying the beta-coefficients for each spectral band of each pixel, and at single bean level, by applying the regression coefficients to the average spectrum of each cocoa bean. In this way, visualisation of fat distribution across the bean can be obtained, and a predicted average fat content. Our previous paper demonstrated the visualisation of HSI calibrations for seeds, showing the advantages of visualising the spatial variability across seeds, or averaging the spectra that belong to single seeds and then applying a calibration such that the average content per seed is obtained (Caporaso, Whitworth and Fisk, 2017).

The second strategy is likely to be more convenient for practical application, while the first one is more of scientific interest because it gives understanding of the possible accumulation of a cocoa constituent across the beans, thus allowing also plant physiology and biochemical studies. The obtained images are termed “chemical images”, which are graded colour images in which the colour indicates the abundance of an attribute, i.e., lipid content in the present case.

## 3 Results and discussion

### 3.1 Reference analysis of fat content

**Fig. 1:**
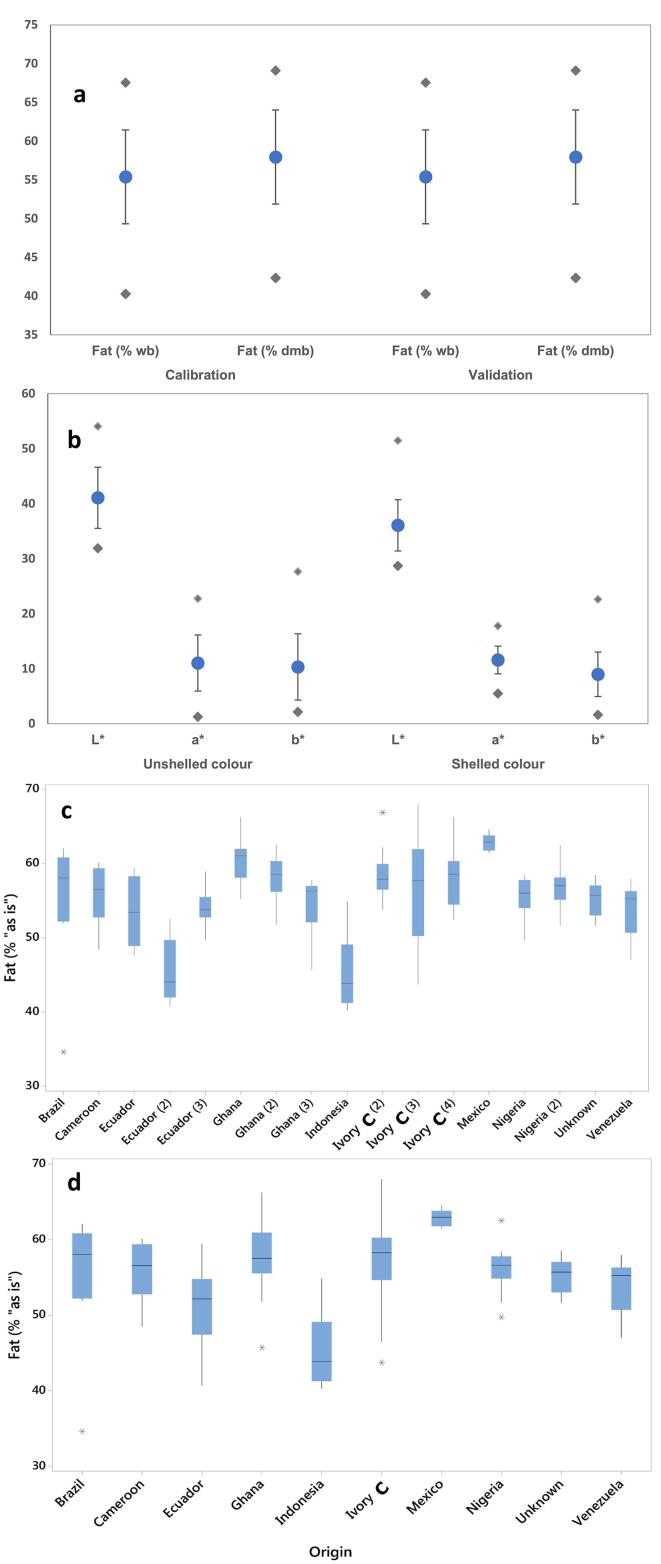
Descriptive statistics for fat content and colour parameters in single cocoa bean levels; (**a**) total fat content in the calibration and validation datasets, (**b**) colour parameters for the unshelled and shelled samples; (**c-d**) variability of total fat in single dry cocoa nibs, expressed on “as is” basis for (c) each batch (n = 10), or (d) grouped by geographical origin. In a-b, circles indicate the mean values, vertical lines indicate the standard deviation and diamonds indicate the range of values.

A preliminary objective was to investigate the natural variability of fat found in single fermented dried cocoa beans. Fig. 1 shows the descriptive statistics of all the parameters analysed, i.e. fat content expressed on “as is” basis and on a dry matter basis (dmb), as well as the colour parameters assessed on the in-shell and the shelled ground nibs. A wide range of lipid content was found in the whole dataset, but also an interesting wide variation of fat content was observed within batches, for which 10 beans were analysed. In a few cases, the fat content was consistent within batches, e.g. for the batch of Mexican cocoa the fat range was below 4% (“as is”), while in the majority of batches the variability was wide, with the maximum variability observed in one batch from Ivory Coast, attaining approximately 25% of the overall range. The observed variability is likely to be due to the interaction between genetics and environment, and great influence is likely to be attributed to the post-harvest conditions, particularly the fermentation and drying steps. Even batches from the same origin, for example from Ivory Coast, had different fat distributions. The median fat content among the three batches from Ivory Coast was almost identical, with very similar average fat content, while their range varied dramatically. This could be due to the different handling of the fermentation process in the three farms, or the use of different agronomical conditions that might lead to higher or lower variability in terms of lipid accumulation in the beans. It should also be recognised that these are commercial samples and, especially in the case of Ivory Coast beans, it is likely that mixing or blending has occurred within the supply chain.

The calibration and independent validation datasets are shown separately (Fig. 1a and Fig. 1b), and a similar range and standard deviation was observed between the two groups, with slightly higher standard deviation for fat content for the calibration dataset, but no statistically significant difference was obtained from a *t*-test (p > 0.05). The fact that the range of fat content for the validation dataset is within the calibration range is desired, and theoretically this should happen also for new samples that will be scanned in the future from new sources, new harvesting years, etc. However, generally, from a practical point of view, NIR and HSI calibrations are intended to be periodically updated with new samples so that new varieties or unexpected samples are correctly classified or quantified.

### 3.2 HSI for fat content prediction

PLS regression models for total fat content of cocoa beans were built from HSI scans of both whole “in-shell” beans and whole nibs, after appropriate treatment of the information in the hypercubes. The results of these prediction models for whole cocoa nibs are reported in Table 1.

**Table 1:** Performance of PLS regression models for total fat content in (a-b) shelled and (c) unshelled single cocoa beans (nibs), and multispectral imaging model based on few selected wavelengths for both sample presentations (d). RMSE = root mean square error of calibration, cross-validation or prediction. RPD = ratio to performance deviation, calculated as the ratio between the reference standard deviation and the RMSECV or RMSEP. MSC = multiplicative scatter correction. SNV = standard normal variate. LV = Latent Variables. Values for the error are indicate as percentage (%, on “as is” or dmb). 238 samples (average spectra) used for calibration, 102 for validation.

**a) Wet basis (“as is”)**	**LV**	**Calibration**		**Cross-Validation**		**Prediction**		**RPD_CV_**	**RPD_P_**
		**R_c_^2^**	**RMSEC**	**R_cv_^2^**	**RMSECV**	**R_p_^2^**	**RMSEP**		
Log(1/R)	6	0.810	2.639	0.781	2.780	0.769	2.785	2.18	2.05
Normalisation	7	0.809	2.710	0.798	2.718	0.824	2.385	2.23	2.40
MSC	6	0.829	2.505	0.842	2.286	0.841	2.273	2.65	2.52
1st derivative	5	0.814	2.609	0.814	2.425	0.809	2.486	2.50	2.30
2nd derivative	6	0.813	2.620	0.811	2.573	0.799	2.552	2.35	2.24
SNV	6	0.829	2.503	0.843	2.313	0.841	2.274	2.62	2.51
SNV + 1st derivative	6	0.840	2.440	0.806	2.680	0.827	2.359	2.26	2.42
**b) Dry Matter Basis**	**LV**	**Calibration**		**Cross-Validation**		**Prediction**		**RPD_CV_**	**RPD_P_**
		**R_c_^2^**	**RMSEC**	**R_cv_^2^**	**RMSECV**	**R_p_^2^**	**RMSEP**		
Log(1/R)	5	0.821	2.563	0.773	2.833	0.800	2.543	2.14	2.24
Normalisation	7	0.822	2.579	0.840	2.316	0.795	2.534	2.62	2.25
MSC	5	0.825	2.555	0.801	2.686	0.828	2.353	2.26	2.42
1st derivative	5	0.810	2.651	0.791	2.602	0.791	2.594	2.33	2.20
2nd derivative	6	0.811	2.657	0.785	2.826	0.782	2.653	2.15	2.15
SNV	6	0.825	2.553	0.816	2.452	0.828	2.355	2.47	2.42
SNV + 1st derivative	5	0.840	2.440	0.813	2.617	0.827	2.359	2.32	2.42

**c) Unshelled beans**	**LV**	**Calibration**		**Cross-Validation**		**Prediction**		**RPD_CV_**	**RPD_P_**
		**R_c_^2^**	**RMSEC**	**R_cv_^2^**	**RMSECV**	**R_p_^2^**	**RMSEP**		
Log(1/R)	9	0.525	4.006	0.446	4.340	0.247	4.940	1.40	1.16
MSC	4	0.388	4.738	0.324	5.001	0.169	5.187	1.21	1.10
1st derivative	8	0.652	3.441	0.504	4.098	0.299	4.801	1.48	1.19
2nd derivative	6	0.623	3.581	0.491	4.182	0.519	4.060	1.45	1.41
SNV+1st derivative	6	0.540	3.753	0.421	4.224	0.195	5.110	1.43	1.12

**d) Multispectral models**	**LV**	**Calibration**		**Cross-validation**		**Prediction**		**N. bands**	**Pre- treatment**
		**R_c_^2^**	**RMSEC**	**R_cv_^2^**	**RMSECV**	**R_p_^2^**	**RMSEP**		
Unshelled	3	0.524	4.102	0.492	4.267	0.358	4.692	5	2nd deriv.
Shelled	5	0.838	2.350	0.834	2.388	0.825	2.382	16	MSC
Shelled	3	0.816	2.506	0.812	2.541	0.849	2.214	4	MSC

For the shelled beans, the results are separately reported by expressing the lipid content on wet (“as is”) basis or dry matter basis (dmb). For the model was built on reference data “as is”, the use of spectral pre-treatment caused very slight improvement in the prediction models, with R^2^ values for the calibration models ranging from R^2^ = 0.81 (Log(1/R)) to 0.84 (SNV + 1st derivative). However, a larger difference was observed for the cross-validation and the external validation (prediction) datasets, where the use of log(1/R) spectra showed worse results in terms of R^2^ and prediction errors. Multiple scatter correction (MSC) and standard normal variate (SNV) had similar prediction performance, as expected, as both are intended to remove light scattering effects. Derivatives also showed good performance, with the first derivative treatment model resulting in a slightly lower prediction error. However, MSC treatment led to the model with the best performance and the highest ratio to performance deviation (RPD), with both cross-validation and prediction R^2^ = 0.842–0.841. Both RMSECV and RMSEP were below 2.3% (as is). Considering the range of natural variability observed even within the same batch, often being 10–20%, an error of approximately 2% for a single bean fat determination is acceptable for screening purposes and sorting of higher/lower fraction.

Similarly, the prediction on dry matter basis (dmb) of shelled cocoa beans achieved a good level of performance, with slightly worse R^2^ values and slightly higher calibration and prediction errors than the prediction made on fat content expressed “as is”. This difference might be due to the incomplete drying of samples using the CEM instrument, which is based on microwave drying. While the fat analysis is based on NMR, which is very accurate in analysing fats, moisture is obtained gravimetrically and the error was likely to be higher as the sample size was very small.

The SNV spectral treatment gave generally the best prediction model for fat content expressed as dmb, with calibration R^2^ of 0.825, cross-validation R^2^ of 0.816 and prediction R^2^ of 0.828. The calibration error was 2.55%, with an external prediction error (RMSEP) or 2.36%.

This demonstrates robustness of the model and reliability for future applications on unknown samples. Generally, this performance allows the application of the calibration for screening purposes and to estimate single-bean fat content. In some cases, this prediction error is comparable to traditional methods for fat content analysis. For example, the AOAC method 922.06 for fat content through acid hydrolysis has standard deviation (SD) ranging from 0.7 to 7.5%, depending on the type of food analysed. Therefore, the method presented here is perfectly acceptable even for quantification purposes, especially given its advantages for 1) single bean analysis; 2) non-destructive measurement; 3) rapidity and 4) operator skill levels with no hazardous chemicals.

The performance of total fat content in unshelled cocoa beans is reported in Table 1**c**. For this model, the whole beans were scanned before any treatment. A weaker performance was observed compared to the shelled beans. The best model was the one using the 2nd derivative pre-treatment, and it showed R^2^ = 0.62 and 0.52 for the calibration and prediction datasets, respectively. The calibration error was 3.58%, while the external prediction (validation) error was 4.06%. The prediction RPD value was 1.41, thus indicating poorer quality for using this model for quantification purposes. This value might appear relatively high when compared to traditional methods for fat content analysis, but the HSI model herein presented has practical applicability. Therefore, it could still be potentially applied for general screening purposes. Even 4% of prediction error might be acceptable considering that in many of the batches, the single bean variability was above 15%. Thus, using HSI would allow identification of seeds with the highest and lowest fat content in a rapid inexpensive way at the reception before any processing step.

For all the models tested, i.e. shelled and in-shell and expressed “as is” or dmb, the paired *t*-test showed a nonsignificant difference between the predicted values and the reference values, at the significance level of 5%. The confidence intervals for those models are the following: 1) shelled, as is: ±0.265; 2) shelled, dmb: ±0.271; 3) in-shell, as is: ±0.390; 4) in-shell, dmb: ±0.388.

**Fig. 2:**
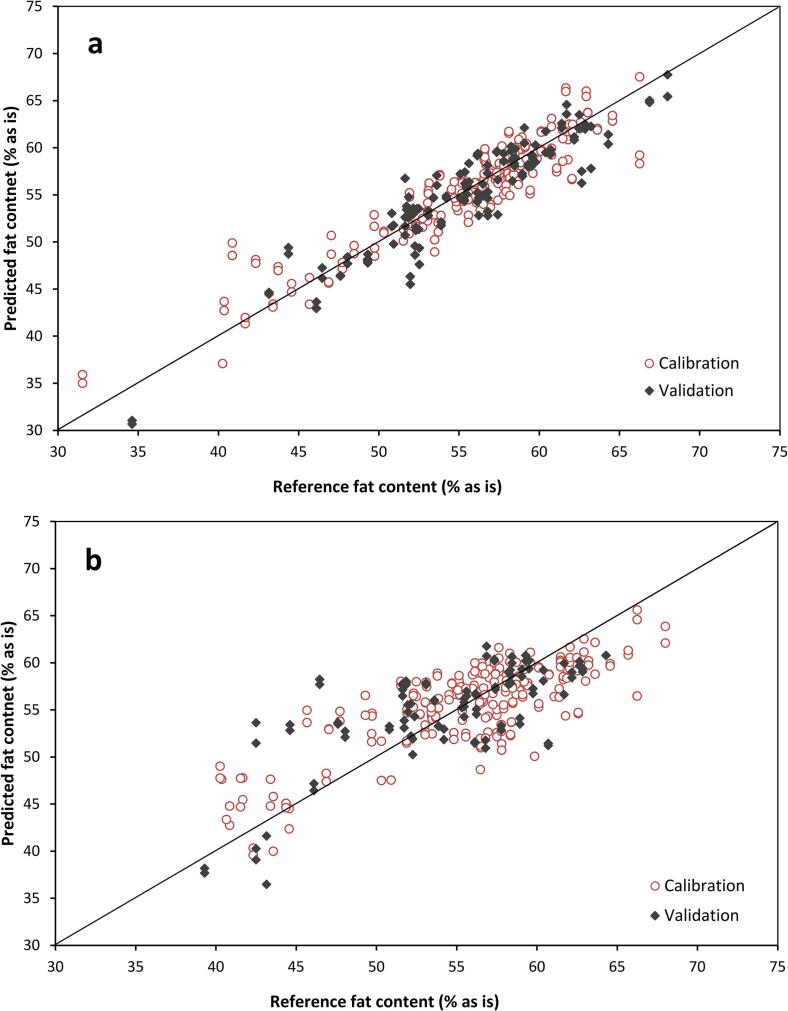
Predicted vs Reference values of fat content in (**a**) whole cocoa nibs and (**b**) unshelled cocoa beans, using the best HSI prediction models.

**Fig. 3:**
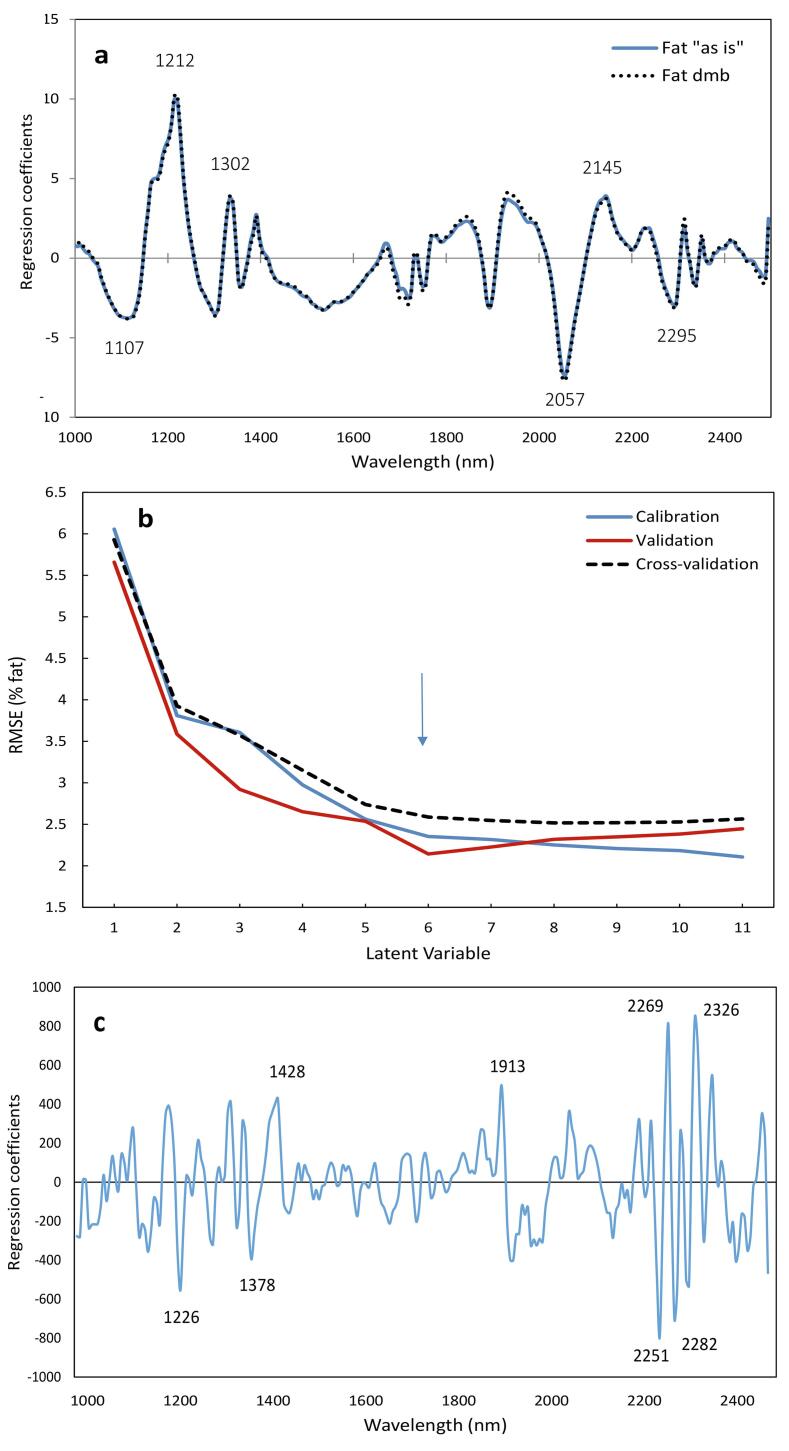
PLS regression model for fat prediction by HSI in single (**a,b**) shelled and (**c**) in-shell cocoa beans. **a**) Regression coefficients for fat expressed on “as is” or dry matter basis for the shelled beans. **b**) Latent Variable plot to express the calibration, cross-validation and external prediction error (RMSE). Models use MSC spectral pre-treatment. **c)** Regression coefficient plot for in-shell beans. Numbers indicate the wavelength in nm. The arrow indicates the selected optimal number of Latent Variables.

Fig. 2 shows the predicted fat content in shelled and unshelled cocoa beans for the best prediction models, while Fig. 3**a,b** reports the β-regression coefficients for these fat quantification models for the shelled beans and Fig. 3**c** shows the models for in-shell beans. The best models using whole cocoa nibs used SNV as the spectral pre-treatment, while the model built on unshelled cocoa beans used 2nd derivative treatment. The wavelengths at 1107, 1212, 1302, 2057, 2145 and 2295 nm were among the most influential ones for the whole cocoa nib models. The regression coefficients of the calibrations were similar for models built on an “as is” basis or on dry matter basis, indicated in Fig. 1a by continuous and dotted line, respectively. Slight differences were observed around 1940 nm, where the O—H bond absorbs strongly. On the contrary, fat prediction model from whole unshelled cocoa beans had major peaks at 1226, 1378, 1428, 1913 and 2250–2326 nm. The most important absorption wavelengths resulting from the regression equation reported by Vaselá et al. (2007) for fat prediction in cocoa powder by traditional NIRS were those at 1728–1744, 2308–2322, 2334–2348 nm. The results herein presented are in agreement with previous literature, as observed here some influence of the bands around 1700 and 2300 nm; however, they are not the most intense ones for the fat prediction model. The calibrations herein presented cannot be directly compared to previous literature, as they used cocoa powder, with fat content ranging from to 5 to ~23% rather than cocoa nibs. In addition, despite the good value of regression coefficient (R^2^ = 0.96), their cross-validation error was 7.0%.

The different performance obtained depending on the spectral pre-processing used can be explained by the different ways in which these treatments remove physical phenomena which are unrelated to chemical information. It is a good practice to test several pre-processing methods to understand the one that brings to the best performance of the multivariate regression model. Whilst it is possible to use raw absorbance spectra to build these calibrations, it is always useful to apply these pre-processing techniques to remove light scattering effects. The most common techniques are Standard Normal Variate (SNV), Multiplicative Scatter Correction (MSC), first and second derivative (better results are achieved when the Savitzky-Golay algorithm), de-trending and normalisation. The SNV treatment effectively removes the multiplicative interferences of scatter and particle size, and the results are similar to those obtained by MSC. Methods such as de-trending and derivatives aim to remove the variation in the baseline, and these spectral pre-processing techniques can be combined to remove unwanted variation in a more effective manner (Rinnan, Van Den Berg and Engelsen, 2009).

The literature is very scarce or non-existent in relation to the application of HSI for qualitative or quantitative prediction of cocoa bean lipid composition, or even on chocolate or other cocoa products, thus a more direct comparison with other chemometric models is difficult. However, other authors applied conventional NIRS to evaluate other parameters in this product, for example sucrose content in chocolate mass (da Costa Filho, 2009), procyanidin content in cocoa liquor (Whitacre et al., 2003), or for the classification of ground cocoa beans from different regions within Ghana by using FT-NIR (Teye et al., 2013).

Previous research reporting on HSI fat calibrations for single peanut kernels demonstrated that the use of the spectral range 1000–2500 nm over the region 400–1000 nm leads to dramatic improvements in the prediction. Indeed, using the visible region led to R^2^ values of 0.536–0.696, depending on the spectral pre-treatment, while the longer wavelength region led to R^2^ values of 0.536–0.923 (Jin et al., 2016). These authors scanned peanut kernels individually by placing only one kernel at a time on the mobile platform (Jin et al., 2016), while in the study herein presented a program was written in IDL + ENVI to manage hypercube processing in a more efficient manner as multiple objects per time can be scanned at a time, thus potentially reducing the acquisition time. In this way, several kernels can be scanned together and contained in the same hypercube. Based on the kernel position in the image, the program was able to attribute a sample number in order to track them individually and export the mean spectra automatically.

### 3.3 Wavelength selection for multi-spectral imaging systems

For applications especially at the industrial level, a multispectral imaging system could be preferred to a full hyperspectral system due to the lower price and lower computational speed requirements. Thus, starting from the full spectrum PLS regression model presented above, a selection of the most important wavelengths was carried out. Selection was made based on the weighted regression coefficients of the PLS regression model. The results of these models are reported in Table 1**d.** For the unshelled beans, the R^2^ value was above 0.5 when using just 5 spectral bands. In the case of shelled cocoa (cotyledons), the performance was much better, with R^2^ values above 0.8. The validation R^2^ value was also above 0.8, when using either 16 bands or 4 bands. Lowering the number of bands did not result in poorer prediction and 4 wavelengths even gave better validation performance (R^2^ = 0.85, RMSEP = 2.2%). This is possibly explained by the strong absorbance bands of lipids, according to the literature (Osborne et al., 1993, Burns and Ciurczak, 2007), and the removal of uninformative bands that bring certain noise in the model. However, it should be pointed out that these models were built on the spectra pre-treated using the second derivative or MSC, which are obviously applicable only when full spectra are available. When using a multispectral imaging system, no spectral pre-treatment is possible anymore, as only a few discrete wavelengths are acquired instead of continuous full spectra, thus only the absorbance data, i.e. log(1/R), can be used. This is likely to bring lower prediction performance due to scattering effects that cannot be correct in multispectral imaging systems.

### 3.4 Application of HSI calibration and visualisation of chemical images

**Fig. 4:**
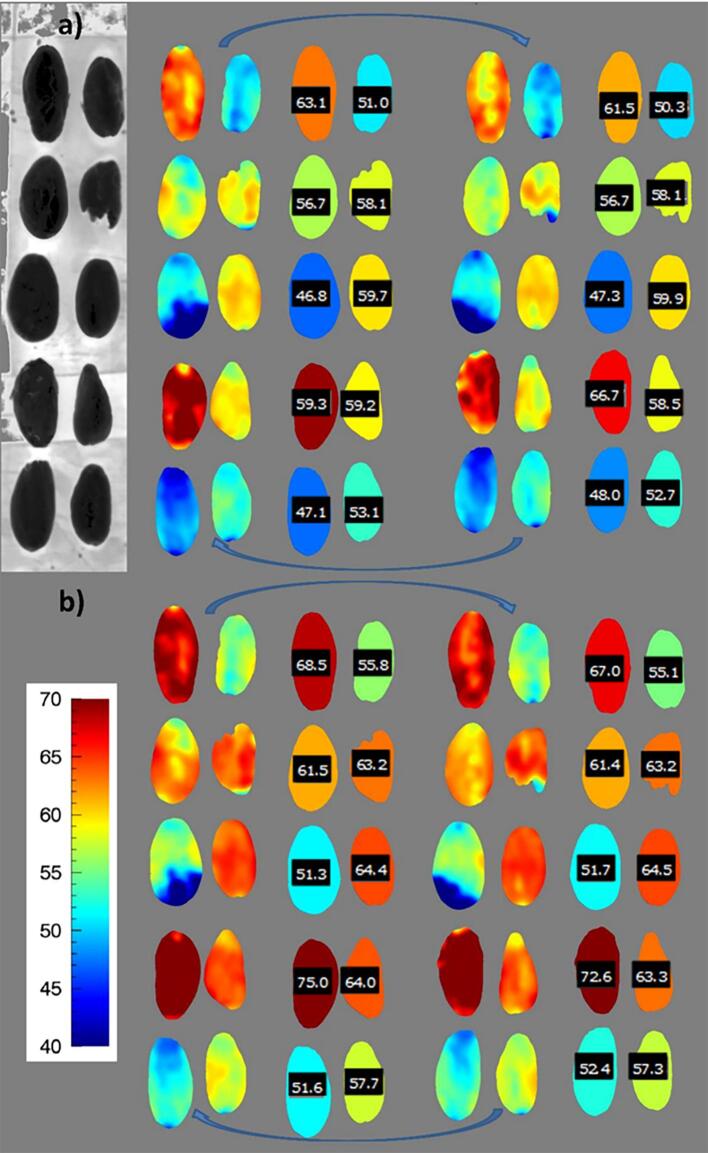
Applied calibration models for total fat content visualisation in unroasted whole cocoa beans (unshelled) at a single pixel level, predicted on (**a**) “as is” or (**b**) dry matter basis. Beans are shown on both orientation, numbers indicate the predicted average value for each bean (batch from Ivory Coast).

The best prediction models based on PLS regression for fat prediction were applied to hyperspectral images of larger numbers of cocoa beans. Once the β-regression coefficients were exported and applied to the hypercubes, it was possible to predict the fat content even at the single pixel level, within each cocoa bean, thus visualising the distribution across the bean. As shown in Fig. 4, the prediction on single pixels allowed visualisation of fat distribution across the beans, as shelled unground beans. Images were acquired on both sides of the beans, by overturning them on the vertical axis (left and right images of Fig. 4, which show the two sides of the beans). Application of the prediction on “as is” basis and on dry matter basis agree, with the expected bias due to the moisture content.

For practicality, it is useful to visualise the predicted fat content as the average for each cocoa bean instead of single-pixel visualisation, thus images were also produced in this sense. This can allow rapid detection of beans with high or low fat content, which can be selected for specific applications, e.g. segregation of beans with low fat content and thus higher non-fat solids, which could be used for dark chocolate manufacture.

**Fig. 5:**
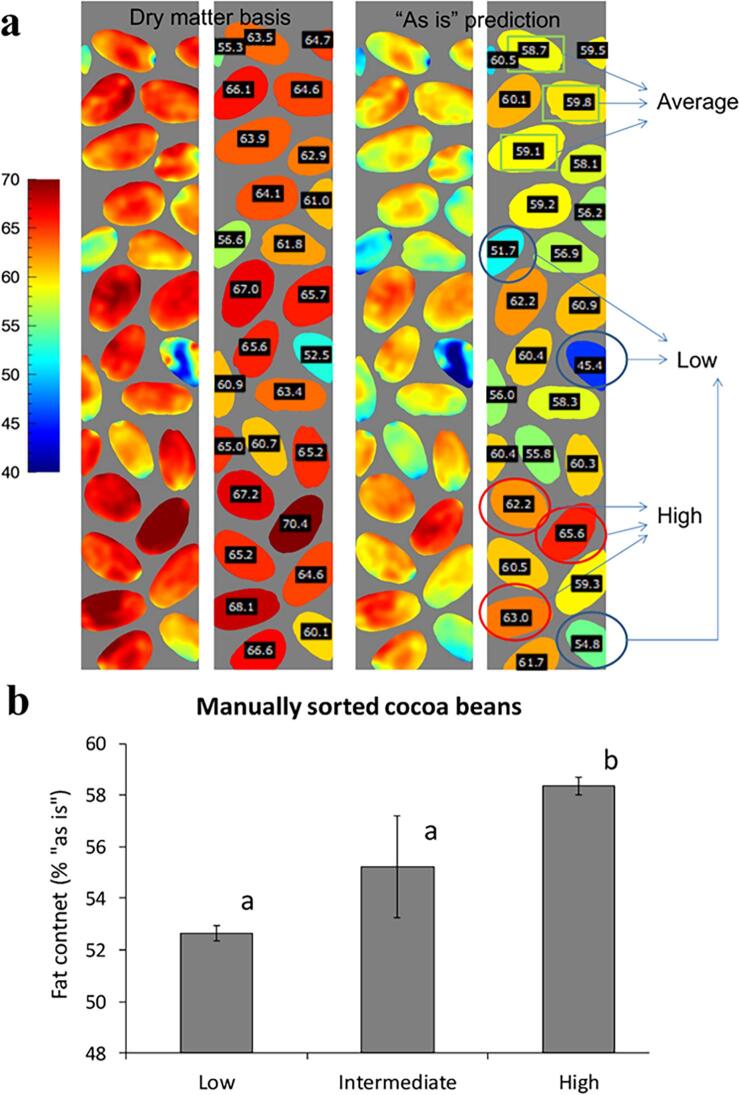
Results of manual sorting of whole cacao nibs for total fat content. **a**) Hypercubes of the scanned beans, shown at ~ 1000 nm. **b**) Average fat content in the three sub-batches, analysed by the reference method (3 beans picked per each batch selected). Bars indicate the standard deviation, and different letters indicate statistically significant differences among the fractions (p < 0.05).

To validate the method and prove the concept of selecting the top and bottom fractions of the cocoa beans based on HSI predicted fat content, a manual sorting experiment was carried out on an independent set of beans, as shown in Fig. 5. Three cocoa beans were picked for the “high fat” fraction, 3 for the “low fraction” batch and 3 belonging to the remaining beans with average fat content (Fig. 4**a**). The beans were manually ground and analysed by the conventional reference method. The results demonstrated that the high and low fractions had statistically significant differences in fat content, with P < 0.01 and with an approximate difference of 6% total lipid content (Fig. 1**b**). The high fraction had also higher fat content than the “average” (batch) fraction, whereas the latter did not show significant difference with the “low” fat fraction, due to the large standard deviation. Therefore, the results showed that it is possible to sort whole cocoa beans into sub-batches, which can be further included in different streams according to the industrial or scientific needs.

## 4 Conclusions

Whilst a few studies previously reported total fat prediction using conventional NIR instruments, previous calibrations were carried out on ground samples and nothing was reported on a single cocoa bean level. The current research therefore established: that (i) within commercial batches of cocoa beans, single beans vary significantly in their fat contents; (ii) that this variation in fat content can be predicted at a single cocoa bean level using HSI; and that (iii) HSI fat content prediction is powerful enough to enable manual sorting of whole cocoa beans, which was demonstrated to enhance the fat content of batches by up to 6%; furthermore (iv) HSI can be used to generate a rough prediction of fat content for the raw cocoa bean even without the need to remove the shell.

## CRediT authorship contribution statement

**Nicola Caporaso:** Conceptualization, Investigation, Methodology, Project administration, Formal analysis, Validation, Visualization, Data curation, Writing - original draft, Writing - review & editing. **Martin B. Whitworth:** Conceptualization, Formal analysis, Methodology, Visualization, Software, Supervision, Writing - original draft, Writing - review & editing. **Ian D. Fisk:** Funding acquisition, Conceptualization, Project administration, Writing - original draft, Writing - review & editing.

## Declaration of Competing Interest

The authors declare that they have no known competing financial interests or personal relationships that could have appeared to influence the work reported in this paper.
