# Supplementary material for: Total lipid prediction in single intact cocoa beans by hyperspectral chemical imaging
Source: Food Chem. 2021 May 15;344:128663. doi: 10.1016/j.foodchem.2020.128663 (PMC7814379; doi:10.1016/j.foodchem.2020.128663)
Supplement: Supplementary data 1 [file mmc1.docx]

ADDITIONAL MATERIAL: (top) Flow chart of the experimental design used for the non-destructive prediction of cocoa bean quality. n=170 refers to the number of beans, while the other numbers indicate the mean spectra (2 spectra per bean). (bottom) List of the cocoa bean samples used in the present experiment.


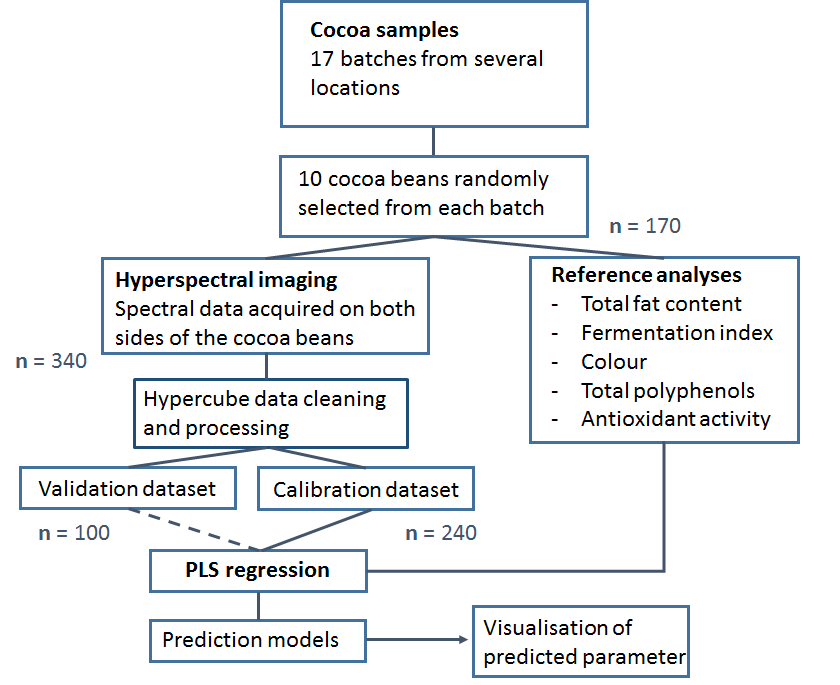


(170 x 2)

| **Sample** | **Country** | **Continent** | **Type / origin of cocoa** |
| --- | --- | --- | --- |
| 1 | n.a. | n.a. | n.a. |
| 2 | Ghana | Africa | Forastero (Amazon hybrids / Amelonado) |
| 3 | Indonesia | Asia | Forastero / Trinitario |
| 4 | Ivory Coast | Africa | Forastero (Amazon hybrids / Amelonado) |
| 5 | Nigeria | Africa | Forastero (Amazon hybrids / Amelonado) |
| 6 | Ecuador | America | Trinitario (also some Arriba Nacional original types) |
| 7 | Cameroon | Africa | Trinitario |
| 8 | Ivory Coast | Africa | Forastero (Amazon hybrids / Amelonado) |
| 9 | Ghana | Africa | Forastero (Amazon hybrids / Amelonado) |
| 10 | Brazil | America | Forastero / Amelonado |
| 11 | Ecuador | America | Trinitario (also some Arriba Nacional original types) |
| 12 | Ivory Coast | Africa | Forastero (Amazon hybrids / Amelonado) |
| 13 | Venezuela | America | Trinitario, possibly some Criollo |
| 14 | Mexico | America | Trintario?, possibly some Criollo |
| 15 | Ghana | Africa | Forastero (Amazon hybrids / Amelonado) |
| 16 | Ecuador | America | Trinitario (and possiblt Arriba Nacional) |
| 17 | Nigeria | Africa | Forastero (Amazon hybrids / Amelonado) |
